# Comparison of the efficacy of dienogest and GnRH-a after endometriosis surgery

**DOI:** 10.1186/s12905-022-02118-w

**Published:** 2023-02-24

**Authors:** Mingjun Tang, Wenhui Yang, Haiyan Zhang

**Affiliations:** Department of Gynaecology and Obstetrics, Baotou Central Hospital of Baotou Medical College, No. 61, Huancheng Road, Donghe District, Baotou, 014000 Inner Mongolia Autonomous Region China

**Keywords:** Dienogest, Endometriosis, VAS score

## Abstract

**Objective:**

To compare the efficacy of dienogest and GnRH-a after endometriosis surgery.

**Methods:**

Patients with endometriosis who were admitted to our hospital from December 2020 to March 2022 were randomly collected. A total of 81 patients were collected and divided into 40 cases in the control group and 41 cases in the observation group. Among them, the control group was treated with GnRH-a drug, and the observation group was treated with dienogest (DNG).

**Results:**

The study found that the therapeutic effects of the two drugs were basically the same in patients with endometriosis. The VAS and Kupperman scores of the control group were 0.78 ± 0.8, 3.9 ± 1.84, *P* < 0.05, respectively; the VAS and Kupperman scores of the observation group were 0.73 ± 0.78, 1.55, respectively ± 1.24, *P* < 0.05, the difference was statistically significant.In the case of postoperative recurrence, the observation group was better than the control group, with 8 cases of recurrence in the control group and 2 cases of recurrence in the observation group, *P* < 0.05.

**Conclusion:**

In the comparison of postoperative efficacy of the two drugs on patients with endometriosis, dienogest is better than GnRH-a adjuvant drug in postoperative recurrence, and has a good improvement and application, which is worthy of further promotion in clinical practice.

## Introduction

Endometriosis is a common gynecological disease, affecting a large number of fertile women worldwide, and its symptoms are also on the rise year by year, which is the main cause of pain and infertility [1–3]. In the course of clinical treatment, the disease usually occurs in the patient’s ovaries, fallopian tubes, cervix and other parts. In the patients who have been found to have endometriosis, nearly 40% of them suffer from infertility, which seriously affecting the normal life of patients [4–6].

It has been found that endometriosis can be treated by surgery, aimed to remove endometriosis lesions to restore the normal anatomy, or by hormone [7]. In the clinical treatment, adjuvant drugs are generally used in patients without cysts found, but with typical endometriosis symptoms, such as intense pain and chronic pelvic pain, treated [8]. For patients with obvious cysts or large cysts, and typical symptoms of endometriosis, surgery is used [9]. At present, the treatment taken for typical endometriosis patients is usually laparoscopic observation and disease diagnosis, adjuvant drugs after surgical treatment, and observing the postoperative response [10, 11]. The drugs commonly used in postoperative treatment include gestrinone, GnRH-a, mifepristone, and so on. After years of clinical research, GnRH-a is recognized as the most prominent drug in the postoperative response and efficacy, and is widely used [12–14]. With the development of medicine, the fourth generation of highly selective progesterone receptor agonist has emerged, and dienogest (DNG) can greatly reduce the adverse reactions of patients, and accurately act on the ectopic lesions in patients [15, 16], and has been proved as the most safe and effective treatment in Asia. Few studies have compared the efficacy of DNG and GnRH-a drugs on endometriosis. This paper compared the efficacy and related adverse effects of GnRH-a and DNG as subsequent adjuvant drugs in endometriosis patients after laparoscopic surgery, to evaluate the feasibility of DNG as a postoperative drug in endometriosis patients, and provide patients with more treatments meeting their own needs.

## Data and methods

### General information

This is a prospective research, 81 patients with endometriosis admitted to our hospital from December 2020 to March 2022 were collected. All patients were treated with corresponding drugs after conservative laparoscopic surgery. The patients were randomly assigned to the control group and the observation group by using a simple random allocation method with a ratio of 1:1. Among them, 40 cases in the control group were treated with GnRH-a after operation; 41 cases in the observation group were treated with DNG after operation.

### Research methods

With full informed consent, the control group was treated with GnRH-a under the guidance of a doctor. GnRH-a of 3.75 mg was injected in the first menstruation of the first month for every 28 days. Three times were a course of treatment. The observation group was treated with DNG. It was started at 3 to 5 days after surgery, with twice every day, and 2 mg every time for 6 consecutive months for 1 course of treatment

### Efficacy evaluation criteria

According to the clinical manifestations of the patients after drug treatment, the curative effect was evaluated. The specific manifestations were sexual intercourse pain, dysmenorrhea, limited uterine activity, vaginal bleeding and other clinical manifestations of the patients. The pain index was evaluated by visual analog scoring system (VAS): draw a 10 cm horizontal line on the paper, with one end of the line being 0, indicating no pain, the other end being 10, indicating severe pain, and the middle part indicating pain of different degrees, Ask the patient to draw a mark on the horizontal line according to their feelings to indicate the degree of pain. 0 is painless, and less than 3 is slight pain, which does not affect sleep; 4–6 points The patients were more painful but tolerable, which affected their sleep. 7–10 patients had severe pain, which affected their sleep seriously; Kupperman rating scale: < 20 points is mild, no need to see a doctor, 20–35 points is moderate, > 35 points is severe, and above moderate needs to see a menopausal clinic in time.

### Observation indicators

1. Age, ASRM score, intraoperative bleeding volume, preoperative symptoms, signs, and preoperative hormone levels of the two groups, and VAS score, Kupperman score, vaginal bleeding, E2 value, FSH value, and recurrence for 3 months and 6 months of follow-up.
2. Results of treatment efficacy evaluation of patients with endometriosis:

Effective: the pain was significantly relieved, and no cyst or mass was found by B ultrasound detection;

Ineffectiveness: the patient’s pain is basically not improved, uterine activity is limited, and cysts or lumps were found with B ultrasound;

Recurrence: After surgery, the pain of the patient is relieved for more than 3 months, or the pelvic mass and tenderness of the patient disappear, and then reappear or worsen to the preoperative level, or the pelvic mass is found in the auxiliary examination (provided that it is within the effective population).

### Statistical methods

Statistical data analysis was performed using the SPSS 23.0 software. Measurement data were expressed using mean ± standard deviation and analyzed with t-sample test. *P* < 0.05 refers to significant difference. The rate is expressed as a percentage. We used the PASS 15 Software (NCSS, LLC. Kaysville, Utah, USA) for sample size calculation. Group sample sizes of 19 and 19 achieve 91.103% power to reject the null hypothesis of equal means when the population mean difference is µ1–µ2 = 3.5–2.0 = 1.5 with standard deviations of 1.5 for group 1 and 1.2 for group 2, and with a significance level (alpha) of 0.050 using a two-sided two-sample unequal-variance *t*-test for the Kupperman score.

## Results

The preoperative conditions of the observation group and the control groups are shown in Table 1. The age of the control group ranged from 25 to 57 years, with a mean age of 40.15 ± 7.68. The observation group ranged from 24 to 59 years, with a mean age of 41.98 ± 7.24. Group *t*-value was − 1.101, *P* > 0.05, which was not statistically significant. The preoperative cyst size of the control group was 1 to 10 cm and the mean cyst size was 5.52 ± 3.01 cm. It in the observation group ranged from 1.7 to 10 cm, and the mean cyst size was 5.51 ± 2.16 cm. The *t*-value between the groups was 0.702, *P* > 0.05, with no statistical difference. The preoperative ASMR score was 27.48 ± 21.73 in control group, and 17.46 ± 14.56 in the observation group. The *t*-value was 2.44, *P* < 0.05, which was statistically significant. The ASMR score results of the endometriosis patients suggested that the patients is basically in the middle stage, and timely clinical treatment is needed.

**Table 1:** General preoperative conditions of the control group and the observation group

Group	Number of sample	Mean age	Cyst size	ASRM score
The control group	40	40.15 ± 7.68	5.52 ± 3.01	27.48 ± 21.73
The observation group	41	41.98 ± 7.24	5.51 ± 2.16	17.46 ± 14.56
*t*		− 1.101	0.702	2.44
*P*		0.063	0.824	0.02

The comparison of VAS and Kupperman score between pre-operation and post-operation in the control group and the observation group are shown in Table 2. The results showed a VAS score of 5.65 ± 2.47 in the control group, and 4.29 ± 2.02 in the observation group before operation, and *t*-value was 0.715 with *P* > 0.05, which were not statistically significant. In the 3-month and 6-month follow-up analysis after treatment, the average VAS score was 0.78 ± 0.8 in the control group and 0.73 ± 0.78 in the observation group. Both of the VAS indicators were basically the same with *P* < 0.05, which was statistically significant. Pre-treatment Kupperman score *P* value greater than 0.05 with no significant difference. After 6 months of follow-up, the mean Kupperman score was 3.9 ± 1.84 in the control group and 1.55 ± 1.24 in the observation group, which were all normal and basically consistent. The above results show that the efficacy of GnRH-a and DNG is basically the same in patients with endometriosis.

**Table 2:** Comparison of VAS scores and Kupperman scores before and after operation

Group	VAS score					Kupperman score				
	Before treatment	3 months after treament	6 months after treament	*t*	*P*	Before treatment	3 months after treament	6 months after treament	*t*	*P*
The control group	5.65 ± 2.47	1.8 ± 1.57	0.78 ± 0.8	3.78	< 0.05	12.4 ± 5.62	3.9 ± 1.84	3.9 ± 1.84	8.9	< 0.05
The observation group	4.29 ± 2.02	1.61 ± 1.48	0.73 ± 0.78	6.73	< 0.05	11.88 ± 5.47	3 ± 1.86	1.55 ± 1.24	10.23	< 0.05
*t*	0.715	3.14	2.47			0.423	2.19	2.53		
*P*	0.477	< 0.05	< 0.05			0.673	< 0.05	< 0.05		

It’s records the basic physical characteristics of both groups during the postoperative treatment in Table 3. As was shown in Table 3, after the treatment with adjuvant drugs GnRH-a and DNG, the days of vaginal bleeding were significantly reduced, and the E2 values and FSH values at 6 months after surgery both reached the normal range, and achieved good efficacy.

**Table 3:** General postoperative conditions of the control group and the observation group

Group	Vaginal bleeding at 3 months after surgery	Vaginal bleeding at 6 months after surgery	E2 value at 3 months after surgery	E2 value at 6 months after surgery	FSH value at 3 months after surgery	FSH value at 6 months after surgery
The control group	4.45 ± 3.01	1.15 ± 1.39	35.23 ± 15.55	43.26 ± 19.29	18.07 ± 9.74	23.07 ± 9.33
The observation group	4.44 ± 3.03	1.15 ± 1.41	68.71 ± 24.14	65.51 ± 33.52	15.58 ± 8.7	19 ± 10.6
*t*	0.02	0.01	− 7.4	− 3.65	1.22	1.8
*P*	> 0.05	> 0.05	< 0.05	< 0.05	> 0.05	> 0.05

It’s records postoperative recurrence after treatment at 6 months in Table 4 records. Six months after postoperative treatment, 8 patients in the control group showed the mass by B ultrasound examination, and only 2 patients in the observation group, which was recorded as the number of recurrence (*P* < 0.05). The table indicate that the recurrence under DNG treatment was less than that GnRH-a treatment in the control group, and the difference was statistically significant.

**Table 4:** Postoperative recurrence in the control group and the observation group

Group	The control group	The observation group	*P*
Recurrence	8	2	0.039
No recurrence	32	39	

It’s records the overall effectiveness of patients at 6 months after surgery in Table 5. The overall effective rate was 77.5% in the control group and 82.9% in the observed group, which remained essentially consistent (*P* < 0.05), and the difference was statistically significant.

**Table 5:** Overall effectiveness of patients after surgery

Group	Effective	Ineffectiveness	Recurrence	Effective rate(%)	*P*
The control group	39	1	8	77.5	< 0.05
The observation group	37	5	2	82.9	

## Discussion

This study shows that the efficacy of DNG and GnRH-a after endometriosis surgery is basically the same, but DNG is relatively superior to GnRH-a in terms of postoperative recurrence. Although the amount of drugs used in the adjuvant treatment of DNG is greater than that of conventional drugs, there is a certain risk of small bleeding, but the two drugs have significantly improved in VAS pain indicators, achieving significant pain relief effect, and achieved good results in vaginal bleeding, E2 value, FSH value after surgery.

A large number of physicians and scholars have conducted a very in-depth study on the causes of endometriosis, and many meaningful views have emerged. Nowadays, many scholars agree with Sampson’s theory, that is, the theory of semen and blood countercurrent implantation. This view holds that the occurrence of endometriosis is due to the fact that the endometrium shed during menstruation flows back from the uterine cavity through the fallopian tube into the pelvic cavity and abdominal cavity, and the glandular epithelium and interstitial tissue in it are ectopic implanted, causing patients to have ovarian, peritoneal and other ectopic lesions [17, 18].

Multiple clinical or surgical treatment factors may influence the risk of postoperative recurrence, but multiple studies have reported inconsistent factors for the risk of postoperative recurrence of endometriosis [19, 20]. In this study, the collected patients were basically similar in terms of operation type, minimizing its impact on postoperative recurrence, and limiting the factors of recurrence to the treatment of adjuvant drugs and the duration of treatment. Through data analysis, the postoperative recurrence rate of DNG was lower than that of GnRH-a. As the fourth generation of highly selective progesterone receptor agonist, DNG can better prevent the recurrence of adverse conditions, such as hot flashes, bone loss, reaching an acceptable degree for patients with less impact on patients’ life [21]. Although the amount of vaginal bleeding reached to abnormal value in some patients during the course of adjuvant therapy, most belonging to irregular vaginal bleeding, the amount of vaginal bleeding has been reduced to varying degrees, and the number of bleeding is also greatly reduced in the extended application of DNG, and after a course of treatment, they basically reached the normal value.

Oral DNG acts precisely on the ectopic lesions. More importantly, the reactivity of DNG in androgen, sugar, mineralocorticoid and other aspects is very low, and the bioavailability can even reach more than 90%, with good tolerance and safety. In contrast, GnRH-a has been widely researched and used in clinical treatment of endometriosis. It can effectively relieve pain, and has also been confirmed in treatment of endometriosis, but low estrogen level caused by DNG can lead to obvious perimenopausal symptoms, seriously affect the patients’ compliance and quality of life. It is more suitable for short-term medication [22]. Actually since surgery is associated to unpleasent complications and reduced efficacy along time, hormonal therapy is the first choice; in patients with contraindications or intollerance to them, pain symptoms resistant to estroprogestinic or progestinic therapy or complicated cases (urinary or bowel occlusion) surgery can be performed in order to ameliorate symptoms and qol [23].

A large number of researchers have tried to explain its pathogenesis, but no single theory can explain all the locations of endometriosis [24]. Although superficial peritoneal lesions and ovarian endometrioma represent the majority of implants for pelvic endometriosis, deep invasive endometriosis and extrapelvic endometriosis are the most challenging situations. Although sometimes drug treatment is sufficient to alleviate symptoms and signs, in a large number of patients, it is necessary to use the method of preserving nerves and blood vessels to completely eradicate, so as to restore the normal pelvic anatomy and function. It has been shown that by inhibiting aromatase and 17β-Hydroxysteroid dehydrogenase type 1 can significantly reduce the estrogen level in endometriotic tissue, increase the apoptosis of endometriotic cells, and reduce the proinflammatory cytokines produced by endometriotic stromal cells [25, 26].

## Conclusion

According to the comparison of the efficacy of DNG and GnRH-a after endometriosis surgery, the two drugs are basically the same in terms of comprehensive efficacy, but DNG is better than GnRH-a in postoperative recurrence. Clinical studies have proved that DNG has higher compliance, low side effects, and better patient tolerance. The treatment of endometriosis with DNG reaches the efficacy level of GnRH-a, and the postoperative recurrence of DNG is better than GnRH-a. However, the treatment mechanism and dosage need further research, and more research from clinicians are needed to determine.

In conclusion, the postoperative adjuvant treatment of DNG in endometriosis patients significantly reduces pain level and recurrence rate and improves the pregnancy level, which has certain promotion significance in clinical practice.

## Data Availability

All data generated or analyzed during this study are included in this published article.
